# Digital Breast Tomosynthesis for Upgraded BIRADS Scoring towards the True Pathology of Lesions Detected by Contrast-Enhanced Mammography

**DOI:** 10.3390/tomography10050061

**Published:** 2024-05-20

**Authors:** Ahuva Grubstein, Tal Friehmann, Marva Dahan, Chen Abitbol, Ithai Gadiel, Dario M. Schejtman, Tzippy Shochat, Eli Atar, Shlomit Tamir

**Affiliations:** 1 Radiology, Rabin Medical Center, Faculty of Medicine, Tel Aviv University, Tel Aviv-Yafo 49100, Israel; talro2@clalit.org.il (T.F.); marvada@clalit.org.il (M.D.); chenbar@clalit.org.il (C.A.); itayg@clalit.org.il (I.G.); dariosch@clalit.org.il (D.M.S.); elia@clalit.org.il (E.A.); shlomitt@clalit.org.il (S.T.); 2 Biostatistics, Rabin Medical Center, Faculty of Medicine, Tel Aviv University, Tel Aviv-Yafo 49100, Israel; tzippysh@clalit.org.il

**Keywords:** digital breast tomosynthesis, contrast-enhanced mammography, breast imaging

## Abstract

Objective: To determine the added value of digital breast tomosynthesis (DBT) in the assessment of lesions detected by contrast-enhanced mammography (CEM). Material and methods: A retrospective study was conducted in a tertiary university medical center. All CEM studies including DBT performed between January 2016 and December 2020 were included. Lesions were categorized and scored by four dedicated breast radiologists according to the recent CEM and DBT supplements to the Breast Imaging Reporting and Data System (BIRADS) lexicon. Changes in the BIRADS score of CEM-detected lesions with the addition of DBT were evaluated according to the pathology results and 1-year follow-up imaging study. Results: BIRADS scores of CEM-detected lesions were upgraded toward the lesion’s pathology with the addition of DBT (*p* > 0.0001), overall and for each reader. The difference in BIRADS scores before and after the addition of DBT was more significant for readers who were less experienced. The reason for changes in the BIRADS score was better lesion margin visibility. The main BIRADS descriptors applied in the malignant lesions were spiculations, calcifications, architectural distortion, and sharp or obscured margins. Conclusions: The addition of DBT to CEM provides valuable information on the enhancing lesion, leading to a more accurate BIRADS score.

**Advances in knowledge:**

- Incorporating DBT into CEM-detected lesions leads to a significant upgrade in BIRADS scores toward the lesion’s true pathology (*p* > 0.0001).
- This was consistent across all readers, with particularly notable differences observed among less experienced readers.
- The primary driver for the score upgrade was attributed to improved margin visibility facilitated by DBT.

## 1. Introduction

Neoangiogenes or neovascularization involves the creation of new blood vessels, which is how malignant tumors facilitate nourishment. Small capillaries within and around the tumor grow and supply nutrients. These capillaries are permeable, allowing for contrast medium administered into the circulation to leak into the tumor, resulting in tumor enhancement under imaging. This principle is the basis for the high sensitivity of breast magnetic resonance imaging (MRI). Contrast-enhanced mammography (CEM) is an emerging imaging tool that allows for the visualization of neovascularization with the intravenous administration of iodinated contrast material [1,2], much like MRI. A dual energy technique is used wherein two exposures—high and low energy—are made in a single acquisition compression, yielding a low-energy image, which resembles a standard mammogram, and a high-energy image. The low-energy image is subtracted from the high-energy image to obtain a recombined image representing the relative distribution of iodine in the breast [3,4,5,6], thereby distinguishing malignant structures from surrounding normal tissue. The reported sensitivity of CEM ranged from 93% to 100%, and specificity ranged from 63% to 88%, demonstrating significant improvement in both when compared with digital mammography (DM). CEM can be an alternative to MRI, although CEM does not enable evaluation of the axilla or other local nodal groups [1,2,3,4,5,6]. 

CEM has been in clinical use in our institute since 2016. It is applied to the diagnosis and analysis of the extent of breast cancer, in addition to intermediate- and high-risk screening and the evaluation of response to neoadjuvant chemotherapy.

Digital breast tomosynthesis (DBT) is an imaging system where the tube head moves in an arc over the breast. Multiple low-dose two-dimensional projection images of the compressed breast from different angels are obtained. The projections are then reformed into a three-dimensional volumetric whole breast, which is then reconstructed into thin-slice (typically 1 mm) images, using a reconstruction algorithm similar to computed tomography (CT). These thin slices diminish confusing overlapping tissues, evident on conventional mammography, reducing mammographic sensitivity especially in dense breasts. Tomosynthesis images can be obtained in conventional craniocaudal (CC) and mediolateral oblique (MLO) projections, as well as in any other orientation. Each slice is parallel to the projection plane at different heights above the detector. The addition of DBT to standard DM has been shown to improve cancer detection while reducing false-positive rates [7,8,9,10]. The benefit of combining DBT with CEM when lesions have already been detected by contrast injection has not been determined.

The aim of this study was to investigate the value of adding DBT to CEM by evaluating changes in Breast Imaging and Reporting Data System (BIRADS) scores assigned to lesions before and after the addition of DBT and to identify factors contributing to these changes.

## 2. Materials and Methods

### 2.1. Setting and Design

A retrospective study was conducted at a single tertiary university medical center. This study was approved by the local institutional review board, which waived the need for informed consent.

### 2.2. Study Population

All CEM studies including DBT acquired between January 2016 and December 2020 were retrieved. The most common reason for performing CEM in our clinic is to evaluate the extent of diagnosed breast cancer. Therefore, to ensure that the readers were blinded to the lesion pathology, only studies in which more than one lesion was detected were included. The readers were blinded to all lesion pathologies. Data on patient demographics, pathologic findings, and findings on follow-up were collected from the digital healthcare records. Lesions were evaluated using all modalities—DBT, 2nd-look ultrasound, and MRI—and categorized as malignant or benign according to the pathology report or 1-year follow-up breast imaging study.

### 2.3. Image Acquisition, CEM and DBT Technique

All CEM scans were performed with a digital mammography unit (Selenia Dimensions, Hologic Inc., Bedford, MA, USA). Iopromide (Ultravist 300, Bayer, Sacramento, CA, USA), at 1.5 mL per kilogram body weight, was injected intravenously using a power injector at a rate of 3 mL/sec. After 2.5 min, the patient was positioned, and all four images (craniocaudal and mediolateral oblique projections of each breast) were acquired within 10 min. For each view, low-energy exposure (28–33 kVp) and high-energy exposure (45–49 kVp) were obtained at the same time in the same compression. For DBT, a conventional X-ray source was used that sweeps along an arc around the breast to acquire multiple two-dimensional digital images.

### 2.4. Image Interpretation and Lesion Assessment

To evaluate the contribution of DBT, four fellowship-trained breast radiologists with 2–25 years of experience, who were blinded to the legions’ pathology or follow-up results, reviewed and scored each lesion detected according to the BIRADS lexicon [7,11,12] descriptors. A BIRADS score was determined for the CEM (including DM) study and for the CEM + DM + DBT study, simultaneously. Each BIRADS score was compared to the lesion category of benign or malignant. Changes in the BIRADS score (including BIRADS 4A-C) with the addition of DBT were classified as follows:

- Score 1—The BIRADS score with the addition of DBT was higher than the CEM-only BIRADS score for malignant lesions or lower than the CEM-only score for benign lesions (i.e., upgraded towards the lesion’s true pathology; more accurate).
- Score 0—The BIRADS score with the addition of DBT was unchanged or lower than the CEM-only BIRADS score for malignant lesions or higher than the CEM-only score for benign lesions (i.e., downgraded away from the lesion’s true pathology; less accurate).

The effect of the size of the lesion on its conspicuity with the addition of DBT was evaluated using cutoffs of 1 cm and 1.5 cm.

### 2.5. Statistical Analysis

Descriptive statistics were used to characterize the cohort. A signed rank test was used to compare the improvement in BIRADS scores with the addition of DBT for all readers. McNemar’s test was used to compare the first and second measurement for each reader. The Wilcoxon rank test was used to analyze the effect of lesion size. All analyses were performed using SAS, version 9.4 (SAS Institute, Cary, NC, USA). A *p* value of <0.05 was considered significant.

## 3. Results

**Figure 1:**
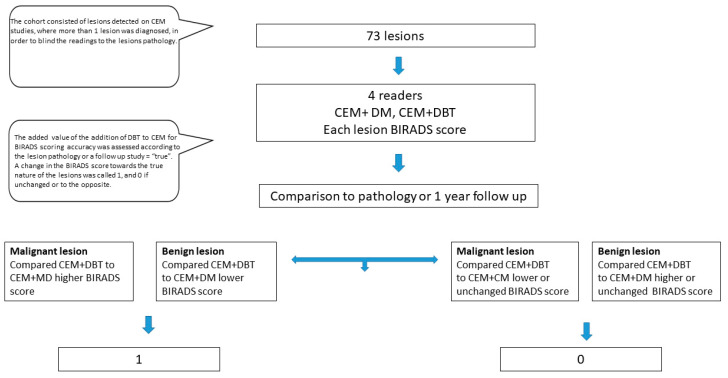
Study flowchart and cohort characteristics.

Figure 1 shows the process of lesion analysis, and Table 1 shows the characteristics of the lesions.

A total of 73 lesions were evaluated, of which 60 (82%) were categorized as a mass. Fifty-five lesions (75%) were malignant; the most frequent pathology (60%) was invasive ductal carcinoma. Of the 18 benign lesions (25%), 8 (44%) were diagnosed by core needle biopsy and 10 (56%) were followed for at least one year. Biopsies performed on 18 CEM-enhancing lesions, 7 under MRI guidance and 11 under ultrasound guidance, yielded 7 malignancies, 4 high-risk lesions, and 7 benign pathologies. The 10 lesions that were detected on CEM and were not biopsied included lesions that were demonstrated on DBT/US/MRI or on CEM only. They were scored as BIRADS 3 and were followed up for at least 1 year (Table 1).

Comparison of the BIRADS scores for lesions detected on CEM before and after the addition of DBT demonstrated a statistically significant change toward the lesion’s actual pathology (score 1) with the addition of DBT (*p* < 0.0001), overall and for each reader. Chi-square values were higher with lower reader experience, implying that the contribution of DBT to the BIRADS score was more pronounced when the reader was less experienced (Table 2). Specifically, there was a change in the BIRADS score toward the lesion’s actual pathology of at least one reader in 62/73 lesions, of three readers in 8 lesions, and of all four readers in 11 lesions. Thus, in 26% of the lesions, the BIRADS scores of at least three of the four readers changed with the addition of DBT. In 15% of the lesions, the BIRADS scores of any of the readers were unchanged or were further away from the lesion’s actual pathology with the addition of DBT. Separate analysis of BIRADS 3 and 4 categories showed that the BIRADS 3 score of two CEM-enhancing lesions was unchanged with the addition of DBT; one BIRADS 4A lesion was down rated to BIRADS 3, two BIRADS 4B lesions were down rated to BIRADS 3, and one BIRADS 4A lesion was down rated to BIRADS 2. All these lesions were benign; the BIRADS scoring was upgraded toward the lesion’s pathology. In 39 malignant and 11 benign lesions, at least one reader changed the BIRADS category toward the actual pathology.

**Figure 2:**
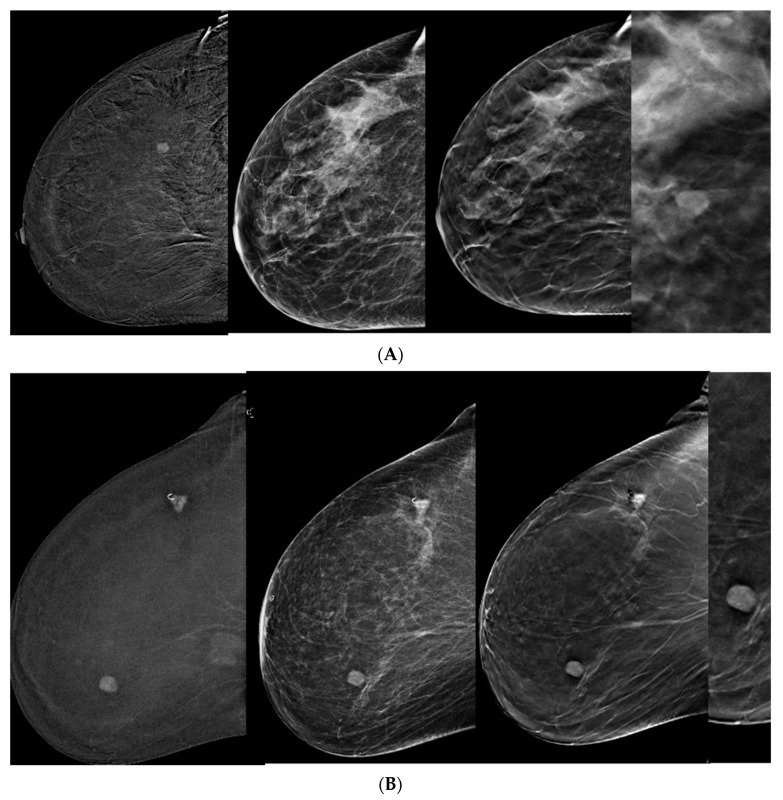

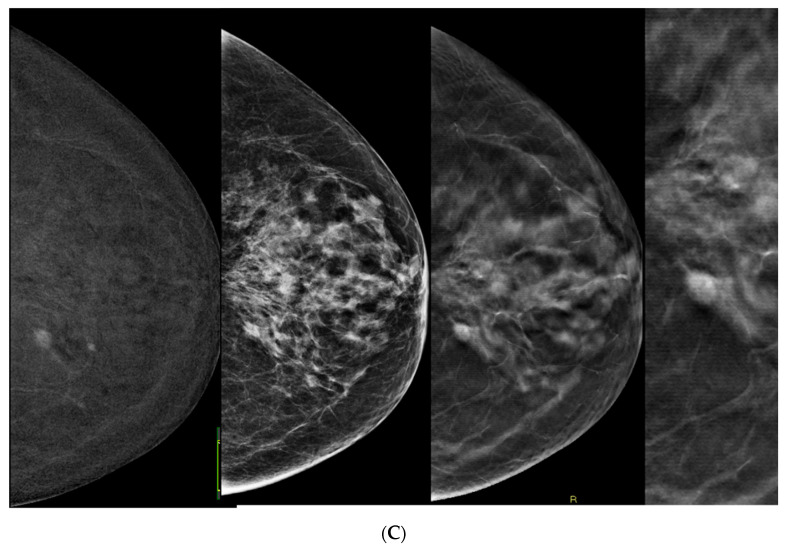
Benign lesions. (**A**) CEM, right craniocaudal (CC) view. Note the enhancing oval mass and regular margins. While not clearly seen on DM, sharp regular margins are visible on DBT and magnified DBT images. (**B**) CEM, right CC view. The known upper outer quadrant breast carcinoma is marked with a clip. Note the additional enhancing mass behind the nipple, regular margins on CEM and DM images, and sharp regular margins on DBT and magnified DBT image. (**C**) CEM, left CC view. Note the oval enhancing mass, non-circumscribed margins not clearly seen on DM, and sharp regular margins seen on DBT and magnified DBT images corresponding to tubular adenoma diagnosed by core biopsy.

**Figure 3:**
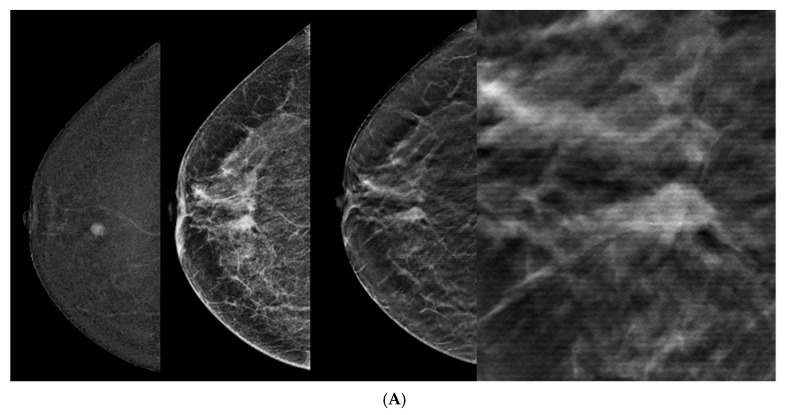

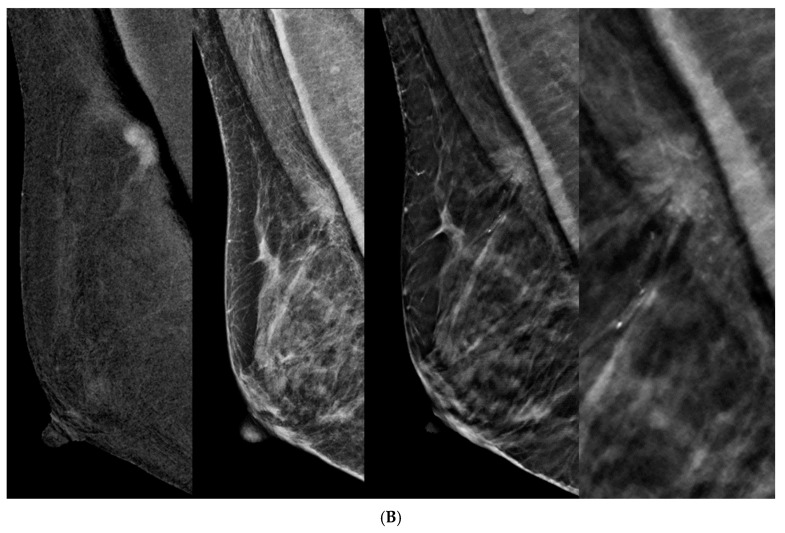
Malignant lesions. (**A**) CEM, right CC view. Note the enhancing oval mass and partially obscured margins not clearly seen on DM. DBT and magnified DBT images clearly demonstrate spiculations, leading to a change to a higher BIRADS score than suggested by CEM alone. The lesion was found to be IDC by pathology. (**B**) CEM, right mediolateral oblique view. Note the enhancing mass, non-circumscribed margins, and increased density on DM image. DBT and magnified DBT images clearly demonstrate spiculations. The lesion is a known IDC.

The main BIRADS descriptors applied in the 55 malignant lesions detected by CEM + DBT were spiculations and architectural distortion in 27 (50%) and calcifications in 7 (13%). Other descriptors were obscured margins, increased density, or their combination. Some of the benign lesions (2/18, 11%) were described as having regular sharp margins (Figure 2 and Figure 3).

The visibility of CEM-enhancing lesions on DBT was affected by size (*p* = 0.0013 using the 1 cm cutoff; *p* = 0.015 using the 1.5 cm cutoff).

## 4. Discussion

CEM is a novel breast imaging modality which holds promise as an alternative to breast MRI using the intravenous injection of a contrast agent for improving the visualization of small lesions that would otherwise go undetected [1,2,3,4,5,6]. Nonetheless, some lesion characteristics are compromised on the subtracted CEM images compared to MRI, including non-well-defined borders due to the lower contrast resolution and lack of time intensity curves, which require dynamic phases. CEM also does not provide data on certain characteristics that can be derived from additional MRI sequences such as T2-weighted images [11,13]. On both CEM and MRI, enhancing the lesion morphology makes an accurate diagnosis possible, regardless of the characteristics of the time intensity curve [11]. A meta-analysis of eight studies evaluating the diagnostic performance of CEM showed an overall specificity of 0.58 with large variability across the different studies [14].

DBT has been shown to have greater diagnostic accuracy than DM for lesion characterization [15]. Wasan et al. [16] reported 97% accuracy in predicting circumscribed lesions as benign when margin sharpness was evaluated by DBT. Others noted a twofold increase in the detection of architectural distortion when DBT was used compared to DM. Most of the distortion was occult on DM and detected only by DBT. Researchers have formulated specific algorithms for the management of DBT-detected architectural distortions [17,18,19].

The performance of combined CEM and DBT was addressed by Petrillo et al. [20] in a study of 134 malignant and benign lesions. There was no significant difference in sensitivity and specificity compared to CEM alone. However, owing to the ability of DBT to differentiate benign from malignant disease and to identify multifocal breast lesions, the authors found the high (91%) sensitivity of the combined technique to be of particular value for detecting and assessing the extent of breast cancer. Huang et al. [21] evaluated 24 lesions in a modified DBT system used to perform CEM. The addition of DBT resulted in better lesion margin assessment by two readers.

The present study showed that margin visibility on CEM + DBT facilitated a BIRADS category change. The assessment of the likelihood of a lesion to be malignant or benign changed significantly for all four readers. This does not contradict the lack of change in sensitivity reported by Petrillo et al. [20] which can be explained by the lesions having already been detected by CEM. The addition of DBT may be assumed to affect mainly specificity, of which the change in BIRADS score is an additional aspect.

Lesion size was associated with lesion visibility on DBT. Reasons for the change in score with DBT included better determination of BIRADS descriptors of margin distortion, spiculations, smooth margins, and calcifications, in accordance with the earlier studies [15,16,17,18,19,20,21]. The change toward the lesions’ true pathology was significant for all readers, who had a wide range of experience. It is noteworthy, however, that it was even more significant for the less experienced readers.

**Figure 4:**
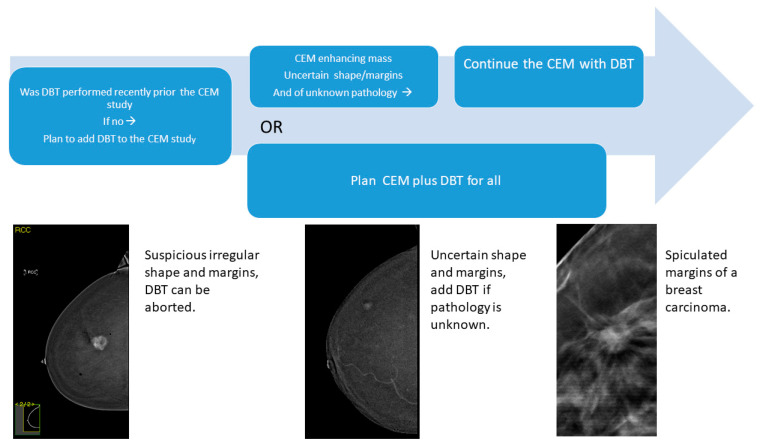
Suggested algorithm for combining DBT with a CEM study.

An algorithm for combining DBT with CEM is suggested in Figure 4. When an enhancing mass demonstrates a definite malignant or benign shape and margin characteristics, or is known from prior workup/pathology, DBT may be aborted. Adding DBT to the CEM study is suggested for an enhancing mass unknown from a prior workup, including additional ipsilateral and contralateral masses detected in diagnostic studies and in screening. The combination of CEM and DBT may have an impact on the screening process as well. For women with dense breasts, CEM, which demonstrated a performance similar to MRI, may serve as a functional examination with DBT for morphology [22]. Concerning radiation exposure, the mean glandular dose is around 2.1 mGy for DM, 2.5 mGy for DBT, and 3.0 mGy for CEM [23]. In a comparison of the addition of DBT to CEM with the addition of spot compression views, Tagliafico et al. [24] reported that the mean glandular dose for DM plus spot compression was 4.69 mGy [24].

Our study has several limitations. A retrospective design was used, and the data were derived from a single center. The cohort included mainly extent-of-disease cases in which lesions were known cancers. Thus, to ensure that the readers were blinded to the pathology and follow-up study results, we restricted the analysis to studies with multiple lesions. To overcome the small sample size, we used multiple readers. There may have been a selection bias, as only studies including CEM and DBT were included. However, at our facility, most CEM studies are conducted simultaneously with DBT unless DBT was recently performed. An additional limitation is the short-term follow-up of the non-biopsied lesions, which was limited to one year.

## 5. Conclusions

In conclusion, the supplementary information provided by adding DBT to CEM is valuable and important. To date, there is no standard recommended protocol for the inclusion of DBT in CEM, highlighting the need for additional research. We believe that adding DBT helped the radiologists feel more confident when addressing lesions for which DM and subtracted images were not sufficient, ultimately providing a more accurate BIRADS score.

## Figures and Tables

**Table 1:** Characteristics of 73 lesions evaluated by CEM + DBT.

Characteristics	Value
Size (mm), mean ± SD	19 ± 23
CEM	
Mass	60 (82%)
Non-mass	13 (18%)
Surgical Pathology	
IDC	44 (60%)
ILC	7 (10%)
Tubular carcinoma	1 (1%)
DCIS	3 (4%)
Fibroadenoma	2 (3%)
Fat necrosis	2 (3%)
FCC	3 (4%)
PASH	1 (1%)
Not biopsied, unchanged on follow-up	10 (14%)
Post-CEM biopsy additional lesions	18 (25%)
IDC	5 (28%)
ILC	2 (11%)
LCIS	1 (6%)
Atypical papillary lesion	2 (11%)
ADH	1 (6%)
FCC	4 (21%)
Tubular adenoma	2 (11%)
Fat necrosis/scar	1 (6%)

Values are expressed as *n* (%) unless otherwise indicated. DBT, digital breast tomosynthesis; CEM, contrast-enhanced mammography; DCIS, ductal carcinoma in situ; IDC, invasive ductal carcinoma; ILC, invasive lobular carcinoma; FCC, fibrocystic changes; PASH, pseudoangiomatous hyperplasia; ADH, atypical ductal hyperplasia.

**Table 2:** BIRADS score change with the addition of DBT.

Score 1 *	*p* Value	Chi Square Value
All readers (years of experience)	<0.0001	
Reader 1 (5 years)	0.0007	16.8919
Reader 2 (2 years)	0.0001	21.0000
Reader 3 (10 years)	0.03	9.2222
Reader 4 (25 years)	0.004	13.3333

* Score 1 = BIRADS score was higher for malignant lesions or lower for benign lesions when DBT was added to CEM compared to CEM alone. DBT, digital breast tomosynthesis.

## Data Availability

Data are contained within the article.

## Abbreviations

BIRADS	Breast Imaging and Reporting Data System
DBT	digital breast tomosynthesis
DM	digital mammography
CEM	contrast-enhanced mammography
MRI	magnetic resonance imaging
